# Artificial intelligence in cardiology

**DOI:** 10.1007/s00508-017-1275-y

**Published:** 2017-10-04

**Authors:** Diana Bonderman

**Affiliations:** Department of Cardiology, Medical University of Vienna, Währinger Gürtel 18–20, 1090 Vienna, Austria

**Keywords:** Decision-making, Cardiology, Artificial intelligence

## Abstract

Decision-making is complex in modern medicine and should ideally be based on available data, structured knowledge and proper interpretation in the context of an individual patient. Automated algorithms, also termed artificial intelligence that are able to extract meaningful patterns from data collections and build decisions upon identified patterns may be useful assistants in clinical decision-making processes. In this article, artificial intelligence-based studies in clinical cardiology are reviewed. The text also touches on the ethical issues and speculates on the future roles of automated algorithms versus clinicians in cardiology and medicine in general.

## Introduction

In modern medicine, decision-making is a complex process that ideally is based on the availability of objective and reliable evidence, immediate access to knowledge, as well as proper interpretation of available facts with the incorporation of patient risk-benefit ratios into every decision step; however, practicing medicine in the real world has taught us that evidence is not always available, penetrance of knowledge takes time and decisions regarding individual patients may not always be objective. In a recent article by Daniel Kahneman (who was awarded the Nobel Memorial Prize in Economic Sciences in 2002) et al. [1], most errors in judgment and decision-making have been attributed mainly to two phenomena, i. e. bias, such as for example stereotyping minorities (social bias) and noise, which means that decisions are influenced by irrelevant factors, such as current mood, time since the last meal and the weather. Taken together, there is clearly room for improvement with respect to generating evidence, structuring knowledge and translating it into clinical decisions.

I strongly believe that incorporation of artificial intelligence tools, such as deep learning and deep reasoning, into day to day medical decision-making will improve patient care. Of course, a prerequisite is that physicians must retain ultimate control, keep an eye on individual decisions and have the authority to override algorithms in clear cut cases.

## Artificial intelligence

Artificial intelligence, in general, is considered a branch of engineering that implements novel concepts to resolve complex challenges. Because biology and medicine are rapidly becoming data-intensive, deep learning algorithms, a collection of automated algorithms that are able to extract meaningful patterns from data collections, have been applied in several fields and have demonstrated breakthrough gains over existing machine learning algorithms. Therefore, a broad implementation of deep learning algorithms in the field of medicine could lead to actionable knowledge and change how we develop treatments, categorize patients, study diseases and make decisions.

In cardiovascular biomedicine, four biomedical big data sources are of particular interest [2]. They encompass 1) functional phenotypes, such as demographics, hemodynamics, electrocardiography, echocardiograms, and imaging data, 2) molecular profiles derived from large-scale panomics data that may be acquired in large trials or the clinical setting, 3) medical records, including patient electronic medical records containing physician’s notes, laboratory test results, and other information on disease, treatment, and epidemiology that may be mined for association studies and predictive modelling on prognosis and drug responses, and 4) literature knowledge: it is estimated that in cardiovascular medicine there is a new publication every 2.7 min. This amount of data overwhelms human intelligence, but may be mined and structured by deep learning algorithms.

## Artificial intelligence in cardiology

Despite its gradual penetration of medicine and biology in general, most cardiologists today are more likely to associate the term artificial intelligence with a futuristic extraterrestrial phenomenon rather than with an engineering tool that is just about to conquer medicine, including cardiovascular medicine.

Only recently, Dawes et al. [3] published a cardiac magnetic resonance imaging-based algorithm of three-dimensional patterns of systolic cardiac motion that enabled them to predict outcome in patients with pulmonary hypertension with high accuracy. Briefly, medical data from 250 patients were used for the study, and the software copied the way more than 30,000 points in their hearts contracted with each beat. This built up a virtual three-dimensional heart for each patient, while the algorithm learned which features were associated with early death or right heart failure.

In an interview, the first author of the study pointed out that one of the most useful functions of using artificial intelligence in this way was: there is no human error. Translating these findings into clinical practice means that physicians are provided with a tool that automatically classifies patients into specific prognosis classes. Compared to the conventional approach, the aforementioned one protects patients from invasive procedures, saves time and manpower and minimizes the grey zone inherent to human judgment.

Another application of deep learning has been impressively demonstrated by Shah et al. [4], who used different algorithms to establish a new phenotypic classification of patients with heart failure and preserved ejection fraction. This clinical syndrome is known to comprise heterogeneous entities and despite its deleterious prognosis none of the treatment strategies tested in numerous clinical trials have so far proven to be effective. It is generally believed that a better phenotyping of affected patients might be the key to successful therapeutic strategies. In a more recent publication, the identified phenogroups (group #1: younger patients with lower B‑type natriuretic peptide levels, group #2: patients with the highest prevalence of obesity and diabetes mellitus, and group #3: oldest patients with the most factors for chronic kidney disease, the most dysfunctional myocardial mechanisms, and the highest adverse outcomes) were confirmed by specific differences in repolarization on their electrocardiograms [5]. This was accomplished by the use of an unsupervised machine learning analysis. Not surprisingly, the findings described have stimulated the field to pursue phenotype-specific research with clinically highly relevant new insights [6–8]. These first successful applications of artificial intelligence algorithms in cardiology will pave the way for more and should be considered as heralds of a new era in cardiovascular medicine.

## Ethical considerations and future perspectives

A broad spectrum of ethical aspects are associated with the practical use of artificial intelligence in medicine and other fields, including transparency in ethical efforts of those who develop artificial intelligence, threat to privacy, threat to human dignity and robot rights. Despite the optimism, we as a society should be aware of the potential threats that are inherent to such powerful tools as artificial intelligence in cases of misuse. Elon Musk, the entrepreneur behind Space-X and Tesla, has recently pointed out that thinking machines might pose an existential danger to mankind and called for regulatory monitoring on national and international levels. The question that we need to ask ourselves as physicians is: how should these warnings be translated into the medical perspective? Our professional ethical codes have a long-standing tradition as documented by the Hippocratic Oath and its modifications as well as more modern codes, e. g. the Declaration of Geneva. We are well advised to follow these, no matter how the medical landscape changes. Our knowledge will grow in parallel with what will be generated by deep learning algorithms. Certainly, human intelligence will not be able to outperform artificial intelligence with respect to knowledge or accuracy in decision-making, provided adherence to professional ethical codes is followed. Developments in robotics may replace practical skills one day; however, our inborn capability to be empathic is probably unbeatable by machines. The eminent theoretical physicist and cosmologist Stephen Hawking recently wrapped up his professional insights by stating that the future of humanity depended on its empathy. In line with this, I strongly believe that the future of cardiology and clinical medicine in general will depend on our empathic skills and the acceptance of artificial intelligence-based assistance tools.

## Conclusion

In the field of clinical cardiology, so far one study has shown that deep learning algorithms clearly outperformed clinicians in predicting prognosis and future events in patients with pulmonary hypertension [3]. In another study, machine learning has helped to develop a clear phenotypic classification of heart failure patients with preserved ejection fraction [4]. Further cardiovascular research based on artificial intelligence tools is underway. Because of its potential to change the way of how we generate knowledge, interpret data and make decisions, artificial intelligence may trigger uncertainties and reservations among healthcare providers and clinicians.

**Fig. 1:**
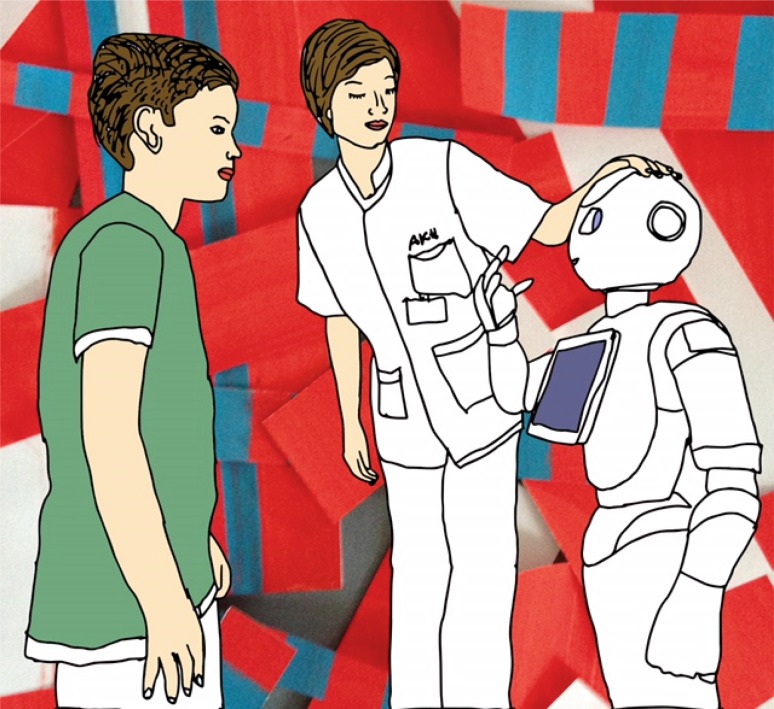
Integration of a communicating humanoid robot in the clinical routine of a cardiology ward. This friendly interface may serve as an icebreaker between clinicians and artificial intelligence algorithms

On the cardiology ward of the General Hospital of Vienna, we are currently testing whether the application of communicating humanoid robots (Fig. 1) as a friendly interface between human and artificial intelligence may facilitate the amalgamation between the world of clinical medicine and smart machines. Whatever the nature of the resulting amalgamation will be, the most powerful tools that clinicians will be able to contribute are our empathy, creativity, and optimism.
